# Supplementary material for: Defective activation and regulation of type I interferon immunity is associated with increasing COVID-19 severity
Source: Nat Commun. 2022 Nov 25;13:7254. doi: 10.1038/s41467-022-34895-1 (PMC9700809; doi:10.1038/s41467-022-34895-1)
Supplement: Supplementary file 1 — Supplemental Information [file 41467_2022_34895_MOESM1_ESM.pdf]

Supplementary Fig 1. Plasma IFNα is consistently reduced with increasing severity in COVID-19 patients. Correlation plots between (**a**) digital ELISA IFNα2 and multi IFNα subtypes (= equivalent IFNα17), (**b**) digital ELISA IFNα2 and Luminex IFNα2, and (**c**) digital ELISA multi IFNα subtypes levels and Luminex IFNα2; first Hopital Cochin cohort, n = 62. Correlation plots between (**d**) digital ELISA IFNα2, (**e**) multi IFNα subtypes, and (**f**) Luminex IFNα2 and days since symptoms. **(g)** IFNβ was measured by digital ELISA in healthy controls (n = 14 donors) and in patients with moderate (n = 15), severe (n = 13) and critical (n = 27) disease. Correlation plots between (**h**) digital ELISA IFNα2 and multi IFNα subtypes (equivalent IFNα17), (**i**) digital ELISA IFNα2 and Luminex IFNα2 levels, (**j**) digital ELISA multi IFNα subtypes and Luminex; second Hopital Cochin cohort, n = 88. Healthy control = black, moderate COVID-19 patients = blue, severe COVID-19 patients = purple and critical COVID-19 patients = red. Rs represents the Spearman coefficient, n= number of individual patients included. Source data are provided as a Source Data file.

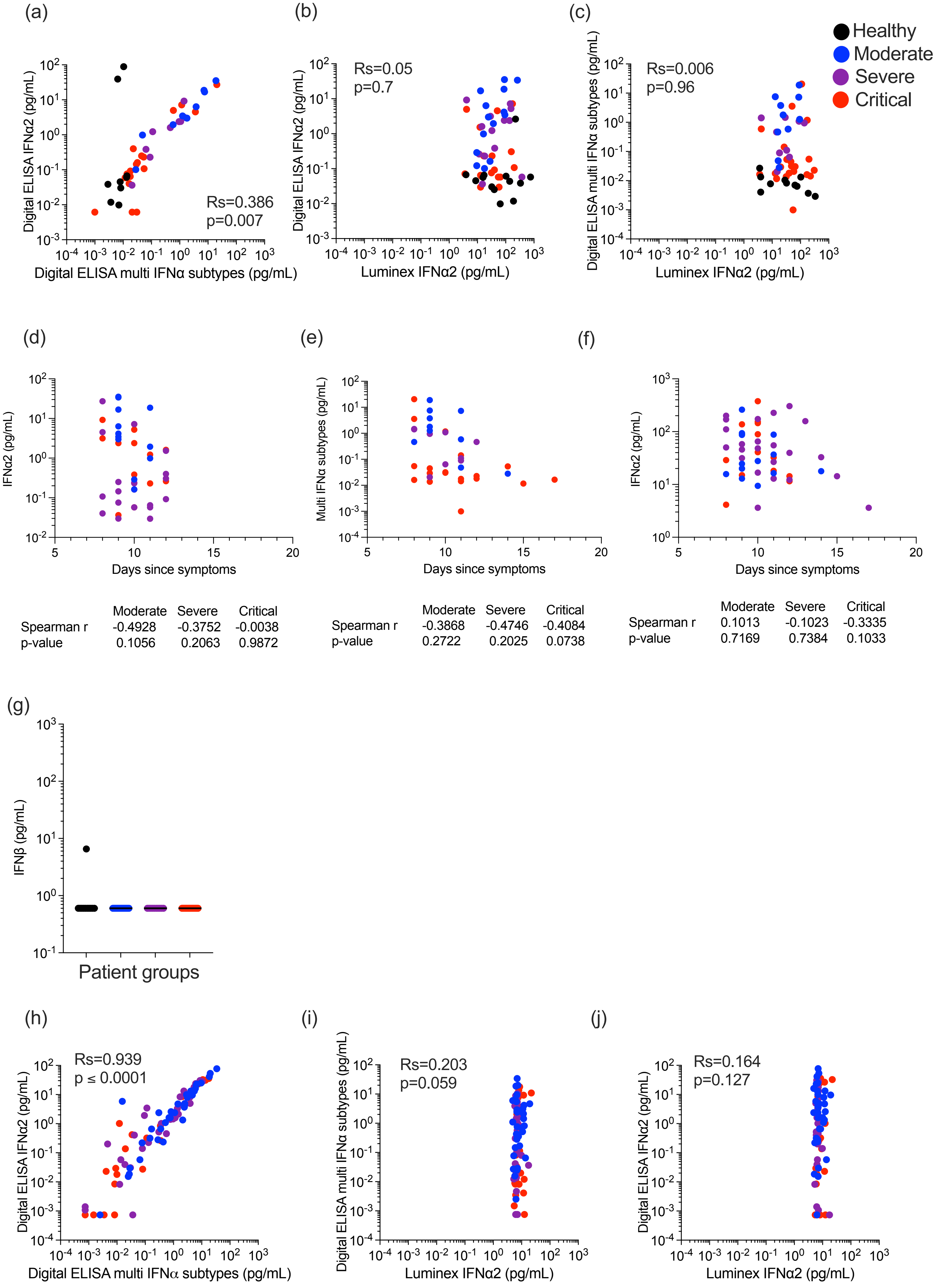

(d)

	Moderate	Severe	Critical
Spearman r	-0.4928	-0.3752	-0.0038
p-value	0.1056	0.2063	0.9872

(e)

	Moderate	Severe	Critical
Spearman r	-0.3868	-0.4746	-0.4084
p-value	0.2722	0.2025	0.0738

(f)

	Moderate	Severe	Critical
Spearman r	0.1013	-0.1023	-0.3335
p-value	0.7169	0.7384	0.1033

Figure S1

Supplementary Fig 2. Plasma IFNα and neutralizing auto-antibodies in COVID-19 patients.

(**a**) IFNβ was measured by digital ELISA in healthy controls (n = 10 donors) and in COVID-19 patients non-hospitalized (n = 46) or hospitalized (n = 80) of the St James Hospital cohort. Correlation plots between ISG zScore and (**b**) digital ELISA IFNα2 (**c**) multi IFNα subtypes and (**d**) IFNβ levels measured by Simoa, n = 50. (**e**) mRNA of IFNA1/13, IFNA14/16, IFNA2, IFNA4/7/10/17/21, IFNA5, IFNA6, and IFNA8 whole blood gene expression in healthy controls, non-hospitalized and hospitalized COVID-19 patients. P values were determined by a Kruskal–Wallis test followed by Dunn's post hoc test for multiple comparisons Correlation plots between anti-IFNα auto-antibodies and (**f**) IFNα2 or (**g**) multi IFNα subtypes digital ELISA levels. (**h**) IFNβ levels as a function of the number of days post symptoms, with regression lines per COVID-19 patient groups shown. Healthy control = black, non-hospitalized COVID-19 patients = green and hospitalized COVID-19 patients = orange. Rs indicates Spearman correlation, n= number of individual patients included. Source data are provided as a Source Data file.

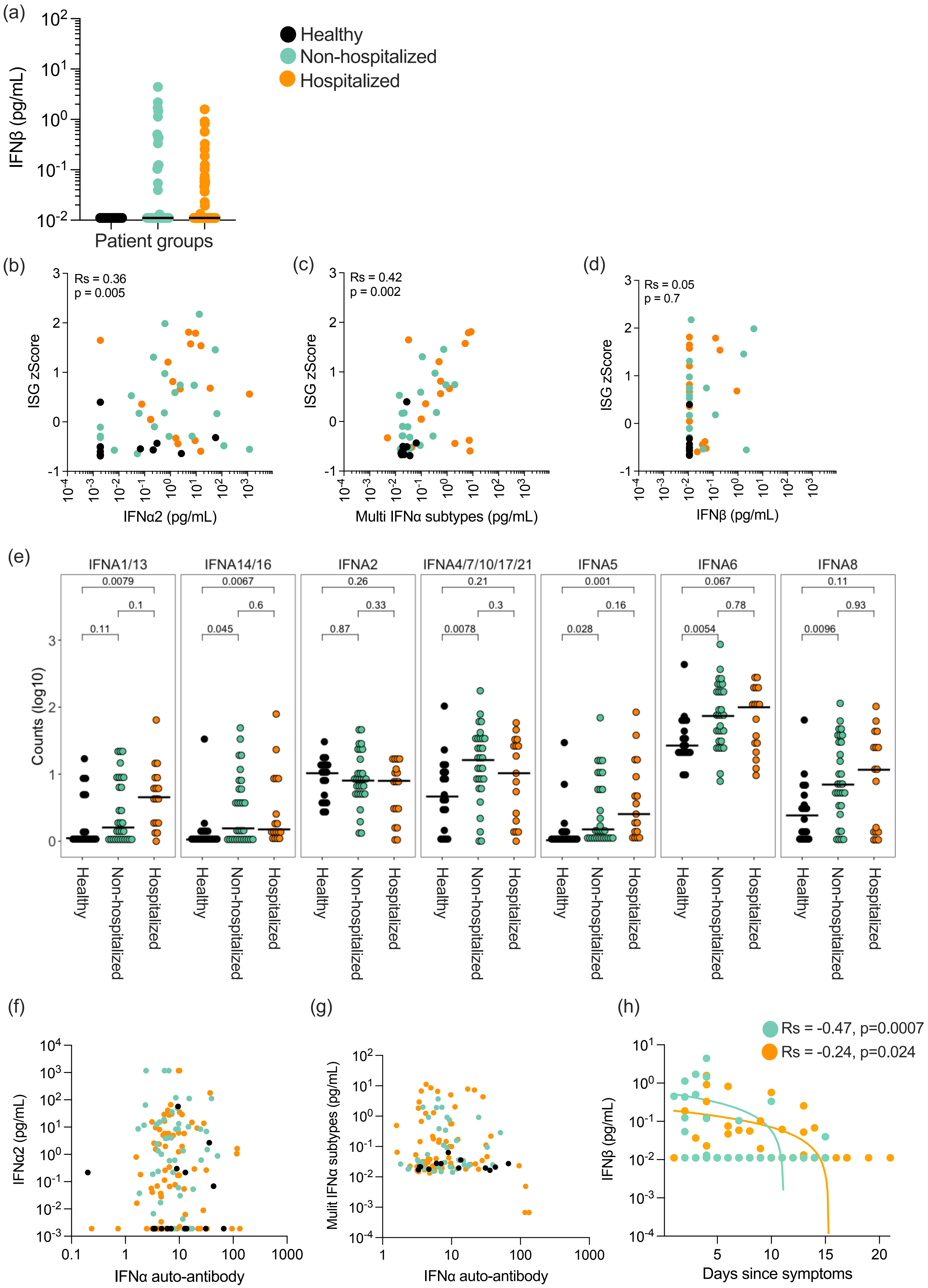
(a)
Healthy
Non-hospitalized
Hospitalized
IFNβ (pg/mL)
Patient groups
(b)
Rs = 0.36
p = 0.005
ISG zScore
IFNα2 (pg/mL)
(c)
Rs = 0.42
p = 0.002
ISG zScore
Multi IFNα subtypes (pg/mL)
(d)
Rs = 0.05
p = 0.7
ISG zScore
IFNβ (pg/mL)
(e)
IFNA1/13
IFNA14/16
IFNA2
IFNA4/7/10/17/21
IFNA5
IFNA6
IFNA8
Counts (log10)
Healthy
Non-hospitalized
Hospitalized
(f)
IFNα2 (pg/mL)
IFNα auto-antibody
(g)
Mulit IFNα subtypes (pg/mL)
IFNα auto-antibody
(h)
Rs = -0.47, p=0.0007
Rs = -0.24, p=0.024
IFNβ (pg/mL)
Days since symptoms

Figure S2

Supplementary Fig 3. Induction of IFN-I response is perturbed in hospitalized patients. GM-CSF (**a**), IL-12p70 (**b**), CXCL10 (**c**), IL-10 (**d**), Granzyme B (**e**), CCL4 (**f**), PDL1 (**g**) and CXCL2 (**h**) were measured by Luminex in healthy control non-hospitalized and hospitalized COVID-19 patients after whole blood stimulation with Poly:IC, LPS and R848. Black lines indicate median values. P values were determined with the Kruskal–Wallis test followed by Dunn's post hoc test for multiple comparisons. healthy control = black, non-hospitalized COVID-19 patients = green and hospitalized COVID-19 patients = orange. N= number of individual patients included. Source data are provided as a Source Data file.

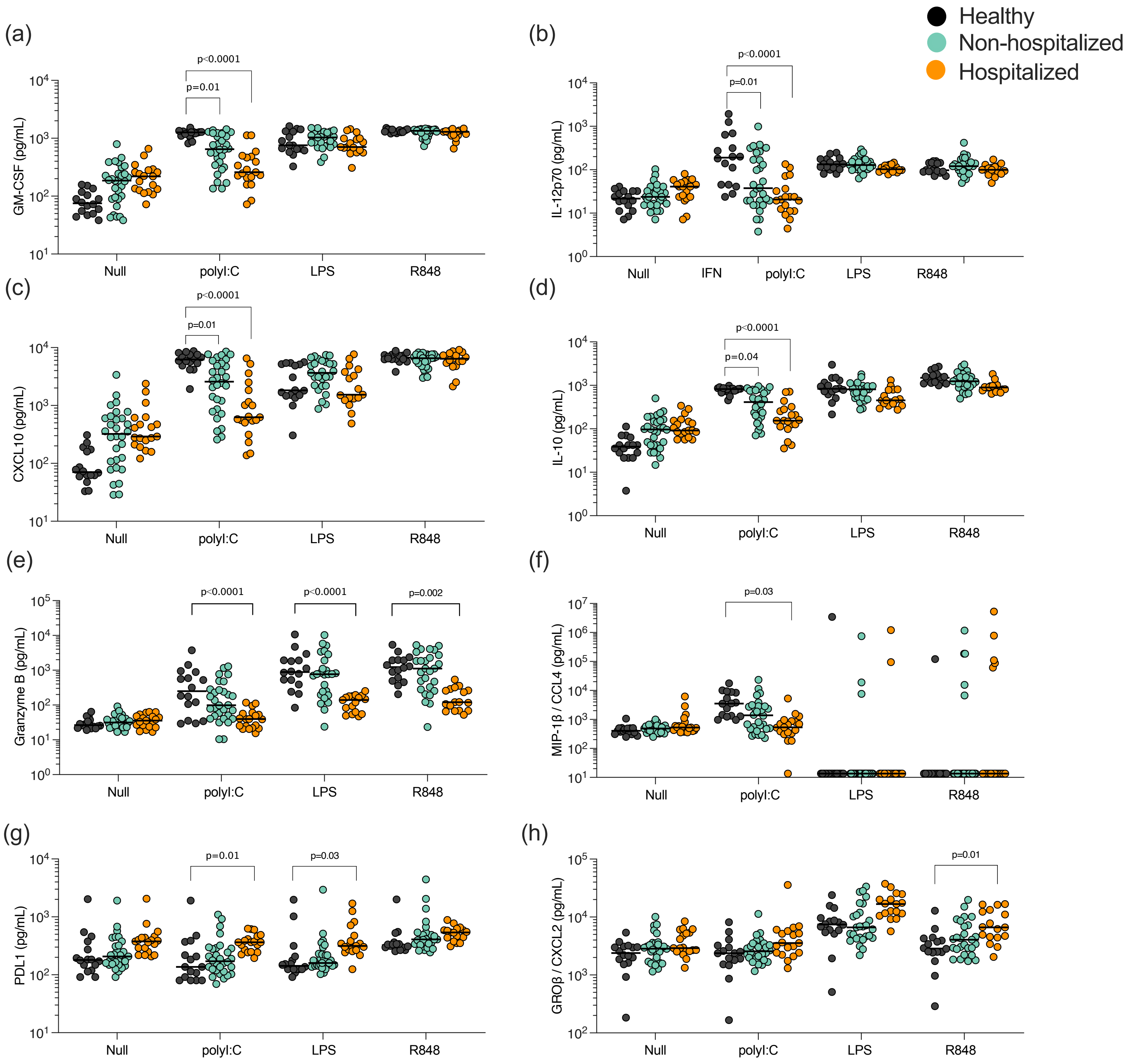

Healthy
Non-hospitalized
Hospitalized
(a)
GM-CSF (pg/mL)
p<0.0001
p=0.01
Null
polyI:C
LPS
R848
(b)
IL-12p70 (pg/mL)
p<0.0001
p=0.01
Null
IFN
polyI:C
LPS
R848
(c)
CXCL10 (pg/mL)
p<0.0001
p=0.01
Null
polyI:C
LPS
R848
(d)
IL-10 (pg/mL)
p<0.0001
p=0.04
Null
polyI:C
LPS
R848
(e)
Granzyme B (pg/mL)
p<0.0001
p<0.0001
p=0.002
Null
polyI:C
LPS
R848
(f)
MIP-1β / CCL4 (pg/mL)
p=0.03
Null
polyI:C
LPS
R848
(g)
PDL1 (pg/mL)
p=0.01
p=0.03
Null
polyI:C
LPS
R848
(h)
GROβ / CXCL2 (pg/mL)
p=0.01
Null
polyI:C
LPS
R848

Figure S3

Supplementary Fig 4. Intra-cellular analysis of pIRF7, pIRF3, and IFNα before and after R848 stimulation. (**a**) Flow cytometry gating strategy. Percentage of positive cells and MFI of (**b**) IFR7, (**c**) IRF3, and intracellular IFNα (**d**) in immune cell subtypes in whole blood of healthy donors without stimulation or after R848 stimulation. Source data are provided as a Source Data file.

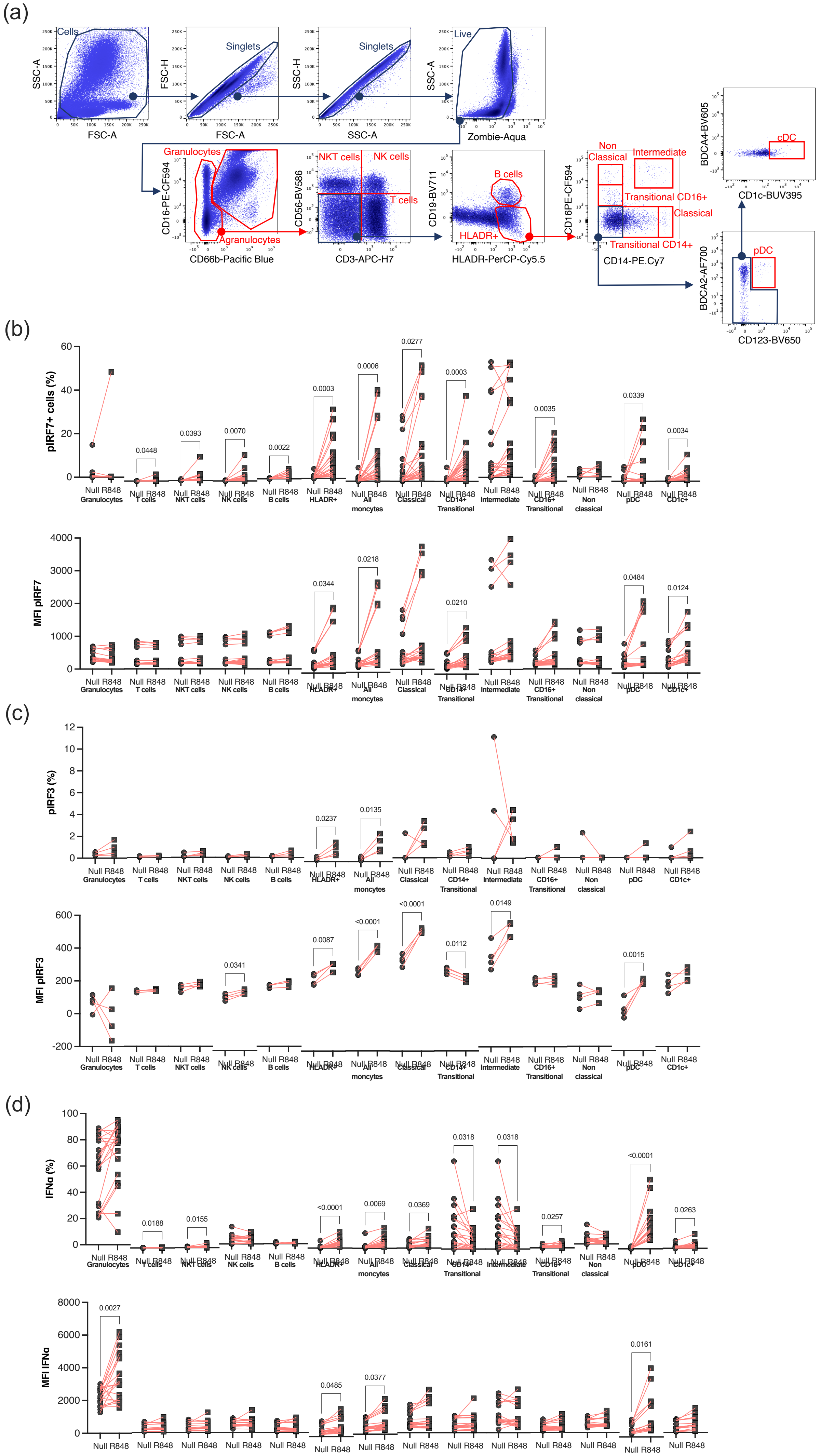
(a)
Cells
Singlets
Singlets
Live
SSC-A
FSC-A
FSC-H
SSC-H
Zombie-Aqua
Granulocytes
Agranulocytes
CD16-PE-CF594
CD66b-Pacific Blue
NKT cells
NK cells
T cells
CD56-BV586
CD3-APC-H7
B cells
HLADR+
CD19-BV711
HLADR-PerCP-Cy5.5
Non Classical
Intermediate
Transitional CD16+
Classical
Transitional CD14+
CD16PE-CF594
CD14-PE.Cy7
cDC
BDCA4-BV605
CD1c-BUV395
pDC
BDCA2-AF700
CD123-BV650
(b)
pIRF7+ cells (%)
MFI pIRF7
(c)
pIRF3 (%)
MFI pIRF3
(d)
IFNα (%)
MFI IFNα
Null R848
Granulocytes
T cells
NKT cells
NK cells
B cells
HLADR+
All moncytes
Classical
CD14+ Transitional
Intermediate
CD16+ Transitional
Non classical
pDC
CD1c+

Figure S4

Table S1 Numbers of patients and healthy controls included in the different immunological analysis

N°	Clinical Cohort	Sample type	Supplemental Data	Healthy controls	Non-hospitalized COVID-19	Hospitalized COVID-19:	(Moderate	Severe	Critical)	Figures	Age range yrs (min-max, median)	Sex (F/M %)
1	Hopital Cochin, Paris	Plasma samples - 1rst cohort	1	14	-	55	15	13	27	Fig 1a-i, S1a-d	26-79, 55	20/80%
2	Hopital Cochin, Paris	Plasma samples - 2nd cohort	2	-	-	89	35	32	21	Fig 1j-l, S1e-g	30-70, 66	31/69%
3	St James, Dublin, Ireland	Plasma samples	3	20	51	85	18	22	45	Fig 2a-f	21-71, 61	54/46%
3	St James, Dublin, Ireland	Whole blood stimulation	4	20	30	18	6	4	7	Fig 3a-d, Fig 5, Fig 6	21-71, 57	51:49%
4	Hopital Cochin and Bichat, Paris	Whole blood stimulation	5	24	-	31	-	11	20	Fig 3e-l	47-89, 73	32:68%
4	Hopital Cochin and Bichat, Paris	Cellular phenotyping	5	29	-	31	-	11	20	Fig 4	47-89, 73	32:68%
Total				63	51	260						

Table S2 Flow cytometry panel applied to analyze intracellular IFNa and pIRF expression

**Target**	**Fluorochrom**	**Clone/reference**	**Company**	**Dilution**
CD3	APC-H7	ref. 560176	BD	1/500
CD19	BV711	ref. 563036	BD	1/500
CD56	BV586	ref. 557747	BD	1/100
CD14	PE.cy7	ref. 561391	BD	1/100
CD16	PE-CF594	ref. 562293	BD	1/100
CD66b	Pacific Blue	ref. 562940	BD	1/500
HLA-DR	PerCP-cy5.5	ref. 339216	BD	1/100
CD1c	BUV395	ref. 742751	BD	1/100
BDCA2	AF700	ref. 354228	Biolegend	1/100
BDCA4	BV605	ref. 743130	BD	1/100
IFNα	PE	REA1013	Miltenyi Biotech	1/100
pIRF7	AF488	K47-671	BD	1/100
pIRF3	AF647	E7J8G	CST	1/100

Table S3 List of nanostring genes differentially induced between Null and IFNa stimulations accross all donors and patients

Genes

CCL8	IL27	TRIM21	KPNB1	TBXAS1
CXCL10	MX2	MARCKS	PSMB9	SELE
IFIT1	CASP5	CCL1	JAK2	KLRB1
RSAD2	AIM2	TAP2	MYD88	ULK1
ISG15	UBE2L6	CCL13	CYSTM1	JUN
IFIT3	CD274	C3AR1	FCGR1A/B	TGFB3
OAS3	DTX3L	IRG1/ACOD1	CASP10	IL5
CXCL11	STAT1	SCARB2	LILRA6	LIF
IFI6	GBP1	NTNG2	P2RX7	IL9R
MX1	IFI27	MT2A	CCL17	ACOX1
HERC5	TRIM22	CCR5	PSMB8	IFNA6
IFI44	IFITM1	CD68	HLA-A	CD276
OAS1	STAT2	CARD17	PLAUR	IL1R2
IFIT2	BST2	CASP1	RNF31	CCL20
CCL2	TLR3	TAP1	MAFB	IL20RB
TNFSF10	CCL7	RIPK2	LTBR	NCR3
OASL	C2	CD69	HLA-DRA	VWF
XAF1	TRIM5	IFITM2	MS4A2	IL17C
IFI35	CCR1	LILRA3	C5	CCL22
IDO1	CCRL2	IRF5	CUL1	IFNL2/3
DHX58	LAG3	CCL18	CXCL16	CXCR2
IL1RN	TRIM6	CCL3/L1/L3	HLA-B	IL1R1
CD80	IFI16	RBCK1	DDIT3	IFNA4/7/10/17/21
OAS2	CTSL	IL34	CD1E	CCR8
TCN2	GBP4	CD86	NLRP3	S100A12
TNFSF13B	ISG20	MEFV	CD40	IL1RL2
IRF7	APOL6	SOCS1	FAS	LTA4H
PARP9	CYP2E1	MLKL	IFNA1/13	PC
EIF2AK2	PLAU	NLRC5	EGLN1	CXCL5
IFIH1	CD38	TNFSF18	ACKR2	IL23A
MS4A4A	IL15	CARD16	CXCL6	JAML
FBXO6	TRIM25	CASP4	IL11RA	PLIN4
TLR7	C3	CGAS	ADORA2A	FCRL4
LAMP3	GBP5	APOBEC3G	SLC11A1	CR1
DDX58	CDKN1A	TMEM140	FCGRT	THBS1
ZBP1	ADAR	TRIM56	ANPEP	ITGAM
IFITM3	IRF9	LILRA5	CCL14	MGAM
	SP100	MS4A7	PSTPIP1	CXCL3
	HAVCR2	HLA-DPA1	ALOX5AP	CCL24
		HLA-DRB	NT5E	MRC1
				ITGAE
				ALPL
				MME
