# Supplementary material for: Defective activation and regulation of type I interferon immunity is associated with increasing COVID-19 severity
Source: Nat Commun. 2022 Nov 25;13:7254. doi: 10.1038/s41467-022-34895-1 (PMC9700809; doi:10.1038/s41467-022-34895-1)
Supplement: Supplementary file 3 — Description of Additional Supplementary Files [file 41467_2022_34895_MOESM3_ESM.pdf]

Description of Additional Supplementary Files

Supplementary Data 1. Patient cohort characteristics and associated data of cohort 1.

Supplementary Data 2. Patient cohort characteristics and associated data of cohort 2.

Supplementary Data 3. Patient cohort characteristics and associated data of cohort 3.

Supplementary Data 4. Patient cohort characteristics and associated data of the sub-cohort 3.

Supplementary Data 5. Patient cohort characteristics and associated data of cohort 4.
