# Supplementary material for: Defective activation and regulation of type I interferon immunity is associated with increasing COVID-19 severity
Source: Nat Commun. 2022 Nov 25;13:7254. doi: 10.1038/s41467-022-34895-1 (PMC9700809; doi:10.1038/s41467-022-34895-1)
Supplement: Supplementary file 9 — Reporting Summary [file 41467_2022_34895_MOESM9_ESM.pdf]

Corresponding author(s): Darragh Duffy

Last updated by author(s): Oct 31, 2022

# Reporting Summary

Nature Portfolio wishes to improve the reproducibility of the work that we publish. This form provides structure for consistency and transparency in reporting. For further information on Nature Portfolio policies, see our Editorial Policies and the Editorial Policy Checklist.

## Statistics

For all statistical analyses, confirm that the following items are present in the figure legend, table legend, main text, or Methods section.

| n/a | Confirmed | |
|---|---|---|
| ☐ | ☒ | The exact sample size (*n*) for each experimental group/condition, given as a discrete number and unit of measurement |
| ☐ | ☒ | A statement on whether measurements were taken from distinct samples or whether the same sample was measured repeatedly |
| ☐ | ☒ | The statistical test(s) used AND whether they are one- or two-sided<br>*Only common tests should be described solely by name; describe more complex techniques in the Methods section.* |
| ☐ | ☒ | A description of all covariates tested |
| ☐ | ☒ | A description of any assumptions or corrections, such as tests of normality and adjustment for multiple comparisons |
| ☐ | ☒ | A full description of the statistical parameters including central tendency (e.g. means) or other basic estimates (e.g. regression coefficient) AND variation (e.g. standard deviation) or associated estimates of uncertainty (e.g. confidence intervals) |
| ☐ | ☒ | For null hypothesis testing, the test statistic (e.g. *F*, *t*, *r*) with confidence intervals, effect sizes, degrees of freedom and *P* value noted<br>*Give P values as exact values whenever suitable.* |
| ☒ | ☐ | For Bayesian analysis, information on the choice of priors and Markov chain Monte Carlo settings |
| ☒ | ☐ | For hierarchical and complex designs, identification of the appropriate level for tests and full reporting of outcomes |
| ☒ | ☐ | Estimates of effect sizes (e.g. Cohen's *d*, Pearson's *r*), indicating how they were calculated |

*Our web collection on statistics for biologists contains articles on many of the points above.*

## Software and code

Policy information about availability of computer code

| | |
|---|---|
| Data collection | Flow cytometry data was collected using FACSDiva software (BD) and FlowJo (Treestar), nanostring gene expression data was collected using nCounter (nanosting), Luminex data collected on a bioplex 200 instrument (Biorad) and Simoa data on a HD-1 (Quanterix) |
| Data analysis | GraphPad Prism (version 9), Qlucore (version 3.5), and R (nmet, M3C packages version 7.3) were used for statistical analysis |

For manuscripts utilizing custom algorithms or software that are central to the research but not yet described in published literature, software must be made available to editors and reviewers. We strongly encourage code deposition in a community repository (e.g. GitHub). See the Nature Portfolio guidelines for submitting code & software for further information.

## Data

Policy information about availability of data

All manuscripts must include a data availability statement. This statement should provide the following information, where applicable:

- Accession codes, unique identifiers, or web links for publicly available datasets
- A description of any restrictions on data availability
- For clinical datasets or third party data, please ensure that the statement adheres to our policy

All manuscript data sets are provided in the supplemental tables specific to each cohort.

## Human research participants

Policy information about studies involving human research participants and Sex and Gender in Research.

| | |
|---|---|
| Reporting on sex and gender | Sex was included in biological analysis as much as possible, as sex is a known factor associated with COVID-19 severity. Sex information on patients is included in the data files. |
| Population characteristics | Age, sex, and clinical status is provided for all donors and patients. No genetic information was collected. |
| Recruitment | Patients were recruited at different clinical centres as described in the methods, based on classical clinical characteristics for defining SARS-CoV-2 infectivity (PCR based test). |
| Ethics oversight | For the Irish studies ethical approval was obtained for the study from the Tallaght University Hospital (TUH)/St James's Hospital (SJH) Joint Research Ethics Committee (reference REC 2020-03). For the French studies samples were obtained from Hopital Cochin and Hopital Bichat, Paris under clinical study protocols in the setting of the local RADIPEM biological samples collection, derived from samples collected in routine care as previously described , or from the INSERM-sponsored French COVID-19 clinical study (NCT04262921). Biological collection and informed consent were approved by the Direction de la Recherche Clinique et Innovation and the French Ministry of Research (no. 2019-3677, 2020-A00256-33). The studies conformed to the principles outlined in the Declaration of Helsinki, and received approval by the appropriate Institutional Review Boards (Cochin-Port Royal Hospital, Paris; no AAA-2020–08018 and Comité de protection des personnes Ile de France VI; no 2020-A00256-33). |

Note that full information on the approval of the study protocol must also be provided in the manuscript.

# Field-specific reporting

Please select the one below that is the best fit for your research. If you are not sure, read the appropriate sections before making your selection.

☒ Life sciences ☐ Behavioural & social sciences ☐ Ecological, evolutionary & environmental sciences

For a reference copy of the document with all sections, see nature.com/documents/nr-reporting-summary-flat.pdf

# Life sciences study design

All studies must disclose on these points even when the disclosure is negative.

| | |
|---|---|
| Sample size | Sample sizes were chosen based on previously published studies on IFN immunity in COVID-19 patients and the expected effect size differences in IFN-I associated phenotypes (Hadjadj et al Science 2020) |
| Data exclusions | Generated data was not excluded, in certain analyses were sample volume was too low certain patients were not included in those specific analyses. |
| Replication | Specific assays were qualified at the point of assay development (prior to this study) to ensure repeatability and reproducibility. For initial findings in one cohort, they were replicated in additional independent cohorts. |
| Randomization | Allocation was not randomized as patients were included in specific groups based on clinical pehnotypes (notbaly requirement for supplmental oxygen). However attempts were made to balance patient groups as much as possible in terms of age and sex. |
| Blinding | Blinding was not possible as samples were labelled by clinicians, and then handled by experimentalists who analyzed the data sets. |

# Reporting for specific materials, systems and methods

We require information from authors about some types of materials, experimental systems and methods used in many studies. Here, indicate whether each material, system or method listed is relevant to your study. If you are not sure if a list item applies to your research, read the appropriate section before selecting a response.

## Materials & experimental systems

| n/a | Involved in the study |
| --- | --- |
| | ☒ Antibodies |
| ☒ | ☐ Eukaryotic cell lines |
| ☒ | ☐ Palaeontology and archaeology |
| ☒ | ☐ Animals and other organisms |
| | ☒ Clinical data |
| ☒ | ☐ Dual use research of concern |

## Methods

| n/a | Involved in the study |
| --- | --- |
| ☒ | ☐ ChIP-seq |
| | ☒ Flow cytometry |
| ☒ | ☐ MRI-based neuroimaging |

# Antibodies

Antibodies used

Target Fluorochrome Clone/reference Company
CD3 APC-H7 ref. 560176 BD
CD19 BV711 ref. 563036 BD
CD56 BV586 ref. 557747 BD
CD14 PE.cy7 ref. 561391 BD
CD16 PE-CF594 ref. 562293 BD
CD66b Pacific Blue ref. 562940 BD
HLA-DR PerCP-cy5.5 ref. 339216 BD
CD1c BUV395 ref. 742751 BD
BDCA2 AF700 ref. 354228 Biolegend
BDCA4 BV605 ref. 743130 BD
IFNα PE REA1013 Miltenyi Biotech
pIRF7 AF488 K47-671 BD
pIRF3 AF647 E7J8G CST
IFNa2 BMS216C eBioscience

Validation

Antibodies were validated for specificity by the supplying company.

CD3 APC-H7 ref. 560176 BD: The SK7 (Leu-4) monoclonal antibody specifically binds to the epsilon chain of the CD3 antigen/T-cell antigen receptor (TCR) complex. This complex is composed of at least six proteins that range in molecular weight from 20 to 30 kDa. The antigen recognized by CD3 antibodies is noncovalently associated with either α/β or γ/δ TCR (70 to 90 kDa).

CD19 BV711 ref. 563036 BD: The SJ25C1 monoclonal antibody specifically binds to CD19, a B lymphocyte-lineage differentiation antigen. CD19, a 90-kDa transmembrance glycoprotein, is a member of the immunoglobulin superfamily and is expressed throughout B-lymphocyte development from the pro-B cell through the mature B-cell stages.

CCD56 BV586 ref. 557747 BD: The B159 monoclonal antibody specifically binds to CD56. CD56 is a heavily glycosylated adhesion protein that is present on a subpopulation of peripheral blood large granular lymphocytes that demonstrate natural killer activity. CD56 is also expressed on a subset of T cells but is not expressed on myeloid cells, erythrocytes or B cells. This antigen is a pan-NK-cell marker. CD56 is virtually identical to an isoform of the neural cell adhesion molecule (NCAM), a structure mediating homotypic and heterotypic cell-cell interactions.

CD14 PE.cy7 ref. 561391 BD: The CD14 antibody, clone MϕP9, is derived from the hybridization of Sp2/0 mouse myeloma cells with spleen cells from BALB/c mice immunized with peripheral blood monocytes from a patient with rheumatoid arthritis. The CD14 antibody binds specifically to the 53–55 kilodalton (kDa) glycosylphosphatidylinositol (GPI)–anchored single-chain glycoprotein, CD14, also known as the LPS receptor or LPS-R.

CD16 PE-CF594 ref. 562293 BD: The 3G8 monoclonal antibody specifically recognizes CD16a and CD16b, low affinity receptors for the Fc region of IgG. CD16a is ~50-65 kDa type I transmembrane glycoprotein that is encoded by FCGR3A (Fc fragment of IgG receptor IIIa) which belongs to the immunoglobulin superfamily. CD16a is also known as Fc-gamma RIII-alpha (Fc-gamma RIIIa or FcγRIIIA) or FcRIIIa and is expressed on natural killer cells, activated monocytes, macrophages, γδ T cells, immature thymocytes, and mast cells. CD16a binds immune-complexed or aggregated IgG and associates with CD247/TCRζ in NK cells and FcεRIγ chains in phagocytes and mast cells to transduce intracellular signals. CD16a functions in antibody-dependent cellular cytotoxicity (ADCC) and other antibody-dependent responses including phagocytosis, cytokine production or mediator release. CD16b is a ~48 kDa glycophosyl-phosphatidylinositol (GPI)-linked form that is encoded by FCGR3B (Fc fragment of IgG receptor IIIb). CD16b is also known as Fc-gamma RIII-beta (Fc-gamma RIIIb or FcγRIIIB) or FcRIIIb and is expressed on neutrophils and activated eosinophils. The extracellular region of CD16b is highly homologous to CD16a. CD16b also serves as a receptor for the Fc region of IgG and can bind immune-complexed or aggregated IgG and may be involved in neutrophil adhesion.

CCD66b Pacific Blue ref. 562940 BD: The G10F5 monoclonal antibody specifically binds to CD66b, also known as Carcinoembryonic antigen-related cell adhesion molecule 8 (CEACAM8). CD66b is a glycosylphosphatidylinositol (GPI) linked protein with a molecular weight of 100 kDa expressed on granulocytes. This molecule was previously clustered as CD67 in the Fourth Human Leucocyte Differentiation Antigen (HLDA) Workshop and renamed CD66b in the Fifth HLDA Workshop. CD66b is a member of the carcinoembryonic antigen (CEA)-like glycoprotein family present on granulocytes and referred to as non-specific crossreacting antigens (NCA). Granulocyte activation induced with soluble stimulators (calcium ionophore, phorbol myristate acetate, N-formylmethionyl- leucyl-phenylalanine) results in release and increased expression of NCA. Findings suggest that these molecules may play a role in phagocytosis, chemotaxis and adherence.

HLA-DR PerCP-cy5.5 ref. 339216 BD: AAnti–HLA-DR is intended for in vitro diagnostic use in the identification of cells expressing the HLA-DR antigen, using a BD FACS™ brand flow cytometer. The flow cytometer must be equipped to detect light scatter and the appropriate fluorescence, and be equipped with appropriate software for data acquisition and analysis.

# Clinical data

Policy information about clinical studies

All manuscripts should comply with the ICMJEguidelines for publication of clinical research and a completedCONSORT checklist must be included with all submissions.

| | |
|---|---|
| Clinical trial registration | NCT04262921 |
| Study protocol | Direction de la Recherche Clinique et Innovation and the French Ministry of Research (no. 2019-3677, 2020-A00256-33) and can be accessed on clinical trials.gov under the reference NCT04262921. Cochin-Port Royal Hospital, Paris; no AAA-2020–08018 and Comité de protection des personnes Ile de France VI; no 2020-A00256-33). Tallaght University Hospital (TUH)/St James's Hospital (SJH) Joint Research Ethics Committee (reference REC 2020-03). |
| Data collection | Clinical cohorts are summarised in Table S1. Healthy controls (n=14) and patients acutely infected with SARS-CoV-2 virus (n=144) were previously described 2 (Table S2, S3), or recruited as inpatients or as outpatients following receipt of a positive SARS-CoV-2 nasopharyngeal swab PCR test at St James's Hospital (SJH) in Dublin, Ireland (n=136) (Table S4, S5) from March-June, 2020. Ethical approval was obtained for the study from the Tallaght University Hospital (TUH)/ SJH Joint Research Ethics Committee (reference REC 2020-03). Severity grades were based on admission and supplemental oxygen requirements at the time of sampling. Moderate patients did not require hospitalization at any timepoint. Hospitalized patients requiring supplemental oxygen via nasal cannula (maximal supplemental oxygen flow of up to 6L/min) were considered severe, with critical disease classified as requiring more than 6L of oxygen per minute, either delivered via high-flow nasal oxygen (Airvo) or a venturi mask, a clinical definition previously defined 3,4. Additional hospitalized patients (severe and critical cases) were also recruited for cellular and functional assays (Tables S6) from Hopital Cochin and Hopital Bichat, Paris under clinical study protocols in the setting of the local RADIPEM biological samples collection, derived from samples collected in routine care as previously described 2, or from the INSERM-sponsored French COVID-19 clinical study (NCT04262921). Biological collection and informed consent were approved by the Direction de la Recherche Clinique et Innovation and the French Ministry of Research (no. 2019-3677, 2020-A00256-33). The studies conformed to the principles outlined in the Declaration of Helsinki, and received approval by the appropriate Institutional Review Boards (Cochin-Port Royal Hospital, Paris; no AAA-2020–08018 and Comité de protection des personnes Ile de France VI; no 2020-A00256-33). Plasma samples were obtained from COVID-19 patients (n=311) for cytokine analysis and for autoantibody analysis (n=146), and whole blood for immune stimulations (n=79) and cellular phenotyping (n=31) from subgroups. Written informed consent was obtained from all study participants. Healthy controls (n=63) were asymptomatic adults, matched with individuals with COVID-19 |
| Outcomes | These were observational clinical studies. Pirmary outcomes for our sub-studies were inclusion of sufficient donors for hypothesis testing. Secondary outcomes were comparison of diverse immune phenotypes. |

# Flow Cytometry

## Plots

Confirm that:

- [x] The axis labels state the marker and fluorochrome used (e.g. CD4-FITC).
- [x] The axis scales are clearly visible. Include numbers along axes only for bottom left plot of group (a 'group' is an analysis of identical markers).
- [x] All plots are contour plots with outliers or pseudocolor plots.
- [x] A numerical value for number of cells or percentage (with statistics) is provided.

## Methodology

| | |
|---|---|
| Sample preparation | Whole blood was retrieved and incubated in PBS containing 2% foetal calf serum and 2 mM EDTA (FACS buffer) for 10 minutes at 37°C. After centrifugation, supernatant was removed, and 1x RBC lysis buffer (Biolegend) was added for 15 min at room temperature. Cells were washed in PBS and then incubated with a viability stain (Zombie-Aqua, BioLegend) for 10 min at 4°C. After washing, the cells were resuspended in FACS buffer and stained with an extracellular mix containing the antibodies shown in Table S6. For intracellular staining, Fixation/Permeabilization Solution Kit (BD Cytofix/Cytoperm) was used according to the manufacturer's protocol. Briefly, the cells were fixed for 10 min at 4°C with 100 µl of the Fixation/ Permeabilization solution and then washed and stained in 100 µl of the BD Perm/Wash Buffer containing the intracellular mix of antibodies for 1 hour at 4°C. |
| Instrument | FACS LSR flow cytometer |
| Software | Data acquisition was performed using FACSDiva software (BD Biosciences, San Jose, CA). FlowJo software (Treestar, Ashland, OR) was used to analyze data. |

| | |
|---|---|
| Cell population abundance | This is described in supplemental Figure S4, the median number of Granulocytes was 4x10.4 cells, of T cells 2x10.4, classical monocyes 5x10.2 and pDCs 10.2 cells per uL/blood. |
| Gating strategy | This is described in supplemental Figure S4. Cells were first gated on SSC-A/FSC-A, then on singlets and live cells. Graulocytes were then defined as CD66b positive, and from the non-granulocytes we identified NK T cells as CD56pos/CD3neg, NK cells as CD56pos/CD3pos, and T cells as CD3pos/CD56neg. From the CD56/CD3 double negs we identified B cells as CD19pos/HLADRpos and myloid cells as HLADRpos/CD3neg. from the Myloid cells we identified non classical monocytes as CD16pos/CD14neg, classical monocytes as CD14pos/CD16neg, and intermediate monocytes as CD14pos/CD16pos. from the CD16neg/CD14neg we identified the pDC population as BDCA2/CD123 double positive, and the cDCs as  CD1c positive. |

☒ Tick this box to confirm that a figure exemplifying the gating strategy is provided in the Supplementary Information.
